# Recent Highlights of Research on miRNAs as Early Potential Biomarkers for Cardiovascular Complications of Type 2 Diabetes Mellitus

**DOI:** 10.3390/ijms22063153

**Published:** 2021-03-19

**Authors:** Agnieszka Bielska, Magdalena Niemira, Adam Kretowski

**Affiliations:** 1 Clinical Research Centre, Medical University of Bialystok, 15-276 Bialystok, Poland; magdalena.niemira@umb.edu.pl (M.N.); adamkretowski@wp.pl (A.K.); 2 Department of Endocrinology, Diabetology and Internal Medicine, Medical University of Bialystok, 15-276 Bialystok, Poland

**Keywords:** diabetes, cardiovascular, complications, miRNA, biomarker

## Abstract

Type 2 diabetes mellitus (T2DM) and its complications pose a serious threat to the life and health of patients around the world. The most dangerous complications of this disease are vascular complications. Microvascular complications of T2DM include retinopathy, nephropathy, and neuropathy. In turn, macrovascular complications include coronary artery disease, peripheral artery disease, and cerebrovascular disease. The currently used diagnostic methods do not ensure detection of the disease at an early stage, and they also do not predict the risk of developing specific complications. MicroRNAs (miRNAs) are small, endogenous, noncoding molecules that are involved in key processes, such as cell proliferation, differentiation, and apoptosis. Recent research has assigned them an important role as potential biomarkers for detecting complications related to diabetes. We suggest that utilizing miRNAs can be a routine approach for early diagnosis and prognosis of diseases and may enable the development of better therapeutic approaches. In this paper, we conduct a review of the latest reports demonstrating the usefulness of miRNAs as biomarkers in the vascular complications of T2DM.

## 1. Introduction

Diabetes mellitus (DM) is a group of chronic endocrine and metabolic disorders characterized by defects in insulin production, secretion, and signaling that are insufficient to maintain the right blood glucose level [1,2]. This serious disease affects increasing numbers of people every year. According to the International Diabetes Federation, 463 million people suffered from diabetes in 2019, and the prognosis suggests that this number will increase to 700 million in 2045 [3]. Type 2 diabetes mellitus (T2DM) is the most common type of diabetes. This complex disease is characterized by insulin resistance when cells are not able to respond properly to a normal level of insulin and a progressive loss of β-cell function [1]. Diabetes can lead to acute or chronic complications (Table 1). Many patients do not have any symptoms of T2DM for a long time, and prolonged exposure to high blood sugar levels dramatically increases the risk of additional complications [4]. Due to numerous clinical consequences associated with diabetes, this chronic disease is linked with shorter life expectancy [5]. Notably, long-term hyperglycemia can result in chronic micro- and macro-vascular complications, and related to them, the diabetic foot, nephropathy, neuropathy, and, finally, heart attack and stroke [6,7,8,9,10].

Recent research has shown that cardiovascular diseases are the leading reasons for morbidity and mortality in diabetes [11,12]. The hyperglycemia intensifies and promotes the glycation of proteins, which is a nonenzymatic attachment of sugars to the free amino groups of proteins that can accelerate the occurrence of serious cardiovascular problems.

Nowadays, there is no reliable tool for prompt identification of the patient at risk of developing T2DM and its cardiovascular complications. Most of the currently used methods can only identify the disease at a later stage. These statements suggest that there is an urgent need to identify early specific biomarkers, which would help to predict individual risk of development of diabetes and its complications.

**Figure 1:**
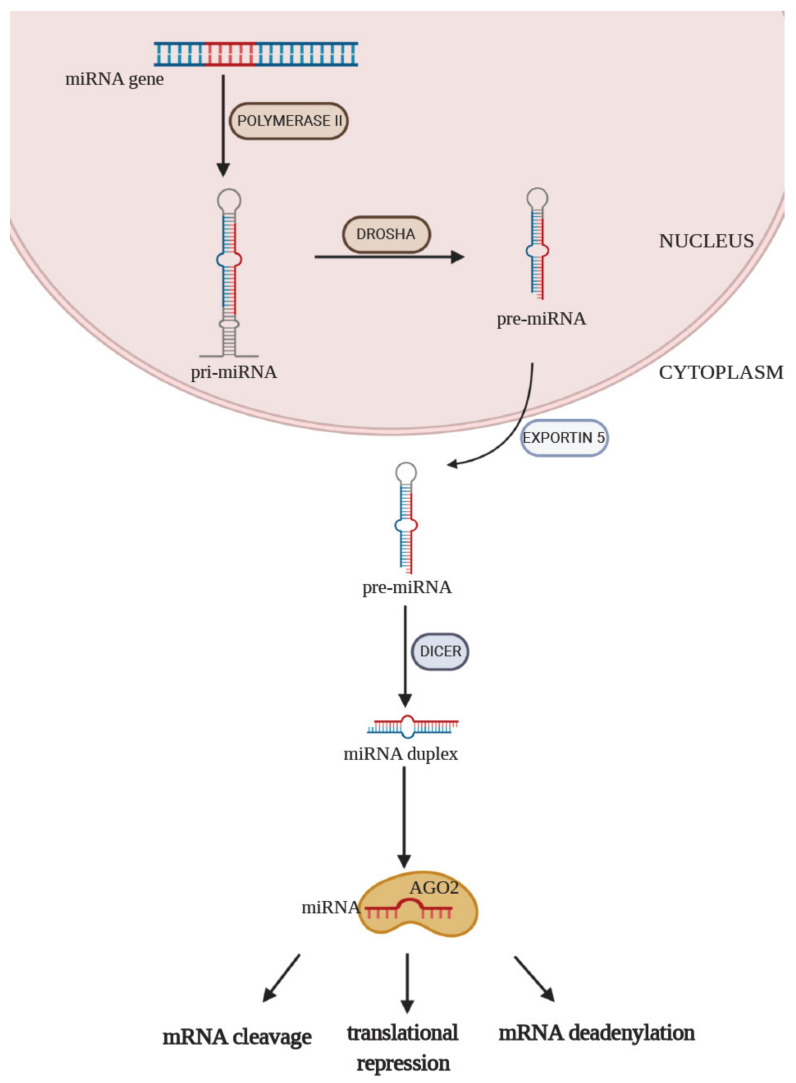
MiRNA biosynthesis and functions. Process of the biosynthesis of miRNA begins in the nucleus. MiRNA genes are transcribed by polymerase II to primary RNA (pri-miRNA). Pri-miRNA is processed to pre-miRNA with the participation of the ribonuclease Drosha. Subsequently, pre-miRNA is transported via Exportin 5 from the nucleus to the cytoplasm of the cell. Dicer is an endonuclease that cleaves pre-miRNAs into short miRNA duplexes, which are later unwound by an unknown helicase. The mature miRNA strand binds to an Argonaute (Ago) protein, forming a complex.

Numerous studies have established that miRNA has tremendous potential to serve as a tool for improving the diagnosis of T2DM, indicate cardiovascular complications at an early stage, or identify patients with a predisposition to develop them. MiRNAs are small (17–25 nucleotides), endogenous, noncoding, single-stranded RNAs, which have a variety of important regulatory effects in cells. MiRNAs play a pivotal role in the regulation of gene expression. They participate in important processes, including cell proliferation, differentiation, and adhesion [13,14]. The control of the expression of targeted genes is achieved by interacting with the 3′ untranslated region (3′UTR) of its target messenger RNA (mRNA). The complementary degree between the miRNA sequence and 3′UTR of its target mRNA determines the regulatory effect of miRNA [15]. MiRNAs are derived from partially complementary primary RNA transcripts (pri-miRNA) produced mainly by RNA polymerase II in the nucleus (Figure 1). To form a precursor of miRNAs (pre-miRNAs), the stem–loop structure of pri-miRNA is cleaved by Drosha and then transported by Exportin 5 to the cytoplasm. At this moment, pre-miRNA is about 70 nucleotides in length. In the next step, a miRNA-specific nuclease Dicer splits it into double-stranded miRNA. In the last step of this process, protein AGO2, which is a member of RISC (RNA-included silencing complex), is involved. One of the strands of miRNA is removed, and the remaining strand is bound to AGO2. This complex targets the 3′ UTR region of mRNA. Association of miRNA with its target mRNA can result in mRNA cleavage, translational repression, or mRNA deadenylation [16,17].

Dysregulation of the expression of miRNAs is associated with the development of various types of cancer, cardiovascular diseases, lung diseases, autoimmune disease, or metabolic disorders. Expression of miRNA is tissue-specific, which allows for identification of its origin [18,19]. MiRNAs have many features of an ideal biomarker. They are stable in biofluids even after a long time after collection and freeze–thaw cycles [20]. The undoubted advantage of these small particles is the availability of material for research. MiRNAs can be collected and detected in a minimally invasive way in easily accessed biofluids, such as serum, plasma, blood, tears, urine, or saliva [14,21]. Despite their numerous benefits, it is also worth paying attention to the limitations of miRNAs as potential biomarkers for various diseases. To avoid misleading results, researchers should strive for normalization and standardization of sample collection, storage, isolation method, and further detection of miRNAs. Interestingly, the level of circulating miRNAs is confounded by age, gender, or some medications [22]. It has been proven that statins decrease circulating miR-122 levels, and heparin (used in medicine as an anticoagulant) influences the PCR reaction during the quantification process [23]. Moreover, some miRNAs are not specific to one disease only [24].

Altered expression of miRNAs can be used as a disease biomarker as well as to understand the pathogenesis. The measurement of the expression levels of miRNA is best carried out with a specific and sensitive assay that allows for the collection of exact data in a short amount of time [25]. Currently, the most commonly used methods for detecting miRNA expression include quantitative reverse transcription PCR (RT-qPCR), different types of microarrays, and next-generation RNA sequencing [21,24]. Identifying the potential targets of miRNAs is crucial for understanding miRNA function. Lately, multiple bioinformatic tools have been developed so that scientists are able to predict possible regulated genes by miRNAs and their functions or their implication in signal pathways [26]. In addition, knowledge that abnormal miRNA expression is associated with many diseases allows for its use as potential therapeutic targets. Manipulation of their expression is used as a new form of clinical treatment [27]. All bioinformatic tools should be validated by scientists to discover novel biomarkers.

**Figure 2:**
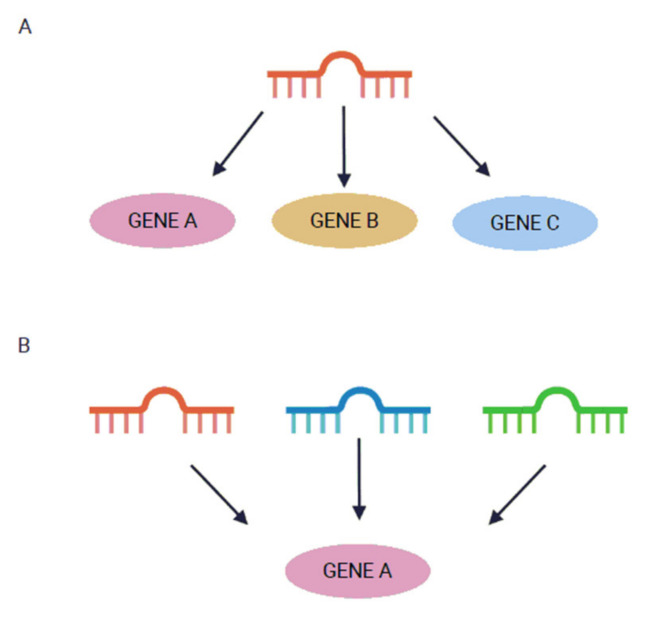
MiRNA binding to target mRNA. A single miRNA can target many mRNAs (**A**). Many miRNAs can target one mRNA (**B**).

Currently, according to the database miRBase (www.mirbase.org, version 22), more than 2600 human mature miRNAs have been identified [28]. Interestingly, it is believed that they are collectively able to regulate about 30% of all genes in the human genome [29]. Bioinformatics prediction suggests that one miRNA could target more than a hundred mRNAs. Conversely, if a single mRNA had complementary sequences for more than one miRNA, it could be regulated by many different miRNAs (Figure 2) [30,31].

Over the last years, several studies on diabetes have indicated specific miRNAs as putative biomarkers for predicting the occurrence of the disease. Interestingly, the dysfunctions of pancreatic β cells composed of the abnormal insulin secretion, and thus impaired glucose tolerance, can occur even 10 years before clinical diagnosis [29,30]. In the prediabetic and diabetic states, expression of miRNAs is altered in organs, but also in biofluids. Many studies have shown that miRNA expression, such as that of miR-375, miR-7, and miR-184, is associated with dysfunction of pancreatic β cells. These miRNAs can play an important role in processes such as proliferation or regulation of crucial pathways [32,33,34]. We know that miRNAs contribute to the regulation of processes related to the proper functioning of pancreatic cells. In addition, many studies argue that these small molecules can serve as a biomarker in detecting T2DM. MiR-126 is one of the most known and the most studied miRNAs in diabetes and its complications. It plays an essential role in endothelial protection and angiogenesis. Interestingly, serum miR-126 was proposed as a biomarker for prediabetes and T2DM [35]. We have previously shown in our study that serum miR-491-5p, miR-1307-3p, and miR-298 can serve as a biomarker for the progression of diabetes. These miRNAs have high diagnostic value for the prediction of T2DM, and we indicated that their expression is dysregulated before T2DM development [36]. MiR-23a seems to be a valuable marker for the early detection of T2DM [37]. Moreover, Pescador et al. found that a three-miRNA panel (miR-15b, miR-138, and miR-376a) is significant for predicting diabetes and obesity [38]. Other interesting findings show that serum-derived miR-486, miR-146b, and miR-15b can also play an important role in predicting the risk of developing DM in obese children. Oerlemans et al. showed in their research that some miRNAs can improve the diagnostic value of currently used biomarkers for acute coronary syndrome identification. MiR-1, miR-499, and miR-21 manifested the significantly increased diagnostic value in combination with hs-troponin T (hs-TnT) (for a combination area under the curve (AUC) = 0.94, whereas hs-TnT alone reached AUC = 0.89) [39].

Levels of certain circulating miRNAs might also be predictive for long-term diabetes complications [25]. The most common complications of DM are vascular pathologies. They are considered as serious manifestations of the disease, and there is an urgent need to understand the pathophysiology of their appearance. This can help with accurate and quick diagnostics and the development of better therapeutic approaches. MiRNAs are the key regulators of cardiovascular system development and maintenance. Altered expression of miRNA in cardiovascular diseases such as arrhythmias, hypertension, myocardial infarction, coronary artery disease, or vascular inflammation is observed [40,41]. In this review, the newest reports regarding the role of miRNA in the vascular complication of T2DM are summarized. In brief, data from a PubMed search were used.

## 2. Vascular Complications of T2DM

T2DM is undoubtedly the cause of numerous vascular diseases, affecting almost all types and sizes of blood vessels [42]. Diabetes vascular complications are divided into microvascular diseases, such as retinopathy (DR), nephropathy (DNP), and neuropathy (DN), and macrovascular diseases, such as coronary artery disease (CAD), peripheral artery disease (PAD), and cerebrovascular diseases including stroke [6,43].

### 2.1. Microvascular Complications

#### 2.1.1. Diabetic Retinopathy (DR)

In DR, two main stages can be distinguished: non-proliferative diabetic retinopathy (NPDR), which usually has no symptoms, and proliferative diabetic retinopathy (PDR) [44], which is the result of damage to the small blood vessels and neurons of the retina in diabetic patients and can lead to blindness [45,46,47]. It is considered that DR might be the most common microvascular complication of diabetes. The risk of developing this disease, as well as other microvascular complications of diabetes, depends on the duration and severity of hyperglycemia [48]. Unfortunately, changes that lead to retinopathy can occur even seven years before the diagnosis of diabetes [49]. Recent studies highlight that miRNA is a very promising tool for the early detection of changes, leading to the development of this pathological state that can help in the prevention of loss of vision in patients with diabetes.

The latest research has shown altered expression of circulating miRNAs in diabetic patients with retinopathy compared to healthy controls and compared to diabetic patients without DR. It was demonstrated that dysregulation of many miRNAs can influence retina cells. Recent studies showing the potential of miRNA as biomarkers for DR are summarized in Table 2.

Rezk et al. in their research manifested a potential diagnostic value of serum miR-126 for DR. In the group of patients with T2DM with DR, they observed significant under-expression of miR-126 compared to diabetic patients without this complication [50]. These results were confirmed in a study by Qin et al., who also suggest that miR-126 might be a suitable candidate for early diagnosis of PDR. The expression of this miRNA was lower in T2DM patients than in the control group. However, the authors did not describe whether patients had T1DM or T2DM [51]. In 2017, Li et al. published a report in which they claimed that miR-200b might target the *VEGFA* gene. Downregulation of serum miR-200b was connected with higher expression of the *VEGFA* gene in the group of patients with DR [52]. The next interesting study indicates a key role of miR-93 in the progression of DR. Elevated plasma miR-93 levels appear to be associated with this complication and can potentially serve as a biomarker. The authors indicate its good diagnostic value in DR using receiver operating characteristic curves (ROCs), where the AUC for miR-93 was 0.866 [53]. About 190 patients with diabetes took a part in the study, which indicated that plasma miR-21 expression was increased in the group of T2DM with DR. ROC analysis for this miRNA showed high AUC values for distinguishing DR patients, indicating the good potential of this miRNA in the diagnosis of this state [54]. The newest work of Liu et al. in the form of a preliminary study indicated that serum-derived miR-221 could be a good potential marker for DR and also for its progression [55]. This study showed upregulation of expression in DR patients and high diagnostic efficiency of miR-221 (AUC = 0.89), which suggest that this miRNA has potential value as a biomarker with more effectiveness than serum angiotensin II (ANG II) and vascular endothelial growth factor (VEGF) (AUC = 0.888 and AUC = 0.785, respectively). In recent studies, it has been suggested that signatures of few miRNAs might be a better predictor for DR than single miRNA. Recent research indicated that serum miRNA signatures consisting of let-7a- 5p, miR-28-3p, and miR-novel-chr5_15976 have good diagnostic value and may serve as a diagnostic biomarker for DR. The calculated AUC of every single miRNA was less than 0.8. Interestingly, a combination of these three miRNAs in one profile showed a significantly higher value of AUC = 0.937 in recognizing diabetic patients with and without DR. Furthermore, the authors of [56] indicated the great utility of this miRNA profile in distinguishing patients in the early stage of the disease from controls without DR. Blum et al. described plasma miR-423 as an important factor that might be involved in PDR. Expression of this miRNA was significantly lower in patients with this disease compared to NPDR, T2DM patients without PDR, and healthy controls. The authors highlight miR-423 correlation with *VEGF*, *NO* (nitric oxide), and *eNOS* (endothelial nitric oxide synthase) expression. This may suggest that changes in the expression of this miRNA are involved in the deterioration of endothelial dysfunction and accelerated atherosclerosis [57]. Pastukh et al. indicated that levels of serum miR-122 are correlated with the severity of DR in T2DM patients. The highest levels of this miRNA were noted in the group of NPDR and the lowest in PDR in comparison to healthy controls and to T2DM patients without retinopathy. This study shed light on the role of miR-122 in DR, but these data should be validated on a larger sample size [58]. Another work indicated that plasma levels of miR-200b were lower in patients with PDR in comparison to patients without this complication [59]. These studies reinforce the importance of further work on miR-200b. RNA sequencing proved that levels of circulating miRNAs from serum might be used as noninvasive biomarkers for early detection of DR. In a recent study, circulating miR-4448, miR-338-3p, miR-485-5p, and miR-9-5p were downregulated, and miR-190a-5p was upregulated in serum samples of DR compared to NDR T2DM patients. Furthermore, bioinformatics validation confirmed that these miRNAs regulate 55 target genes that are mainly connected with the regulation of vascularization processes. Interestingly, miR-9-5p may regulate 41 genes from these targets, which shows the great potential of this miRNA in further analysis [60]. A recent study described miR-3197 and miR-2116-5p as good potential biomarkers for DR. Furthermore, this paper presents notch homolog 2 (*NOTCH2*) as a possible target gene of miR-2116-5p. This gene can be expressed in the retina and regulate *VEGF* gene. ROC analysis showed that the combination of these two miRNAs provides a high AUC score (AUC = 0.972) [61]. Elevated plasma miR-320a expression can differentiate a DR group from diabetic patients without this condition. Interestingly its targeted genes are involved in biological processes significant for DR development. This important report demonstrated that miR-320a plays an important role in the pathogenesis of this complication of T2DM [62]. Smit-McBride et al. conducted comprehensive studies on the miRNA profile in aqueous humor, plasma, and vitreous. They found four miRNAs differently expressed in patients with DR compared to healthy controls. Signatures of let-7b, miR320b, miR-762, and miR-4488 might serve as potential biomarkers for DR prognosis and diagnosis [63].

Some studies show changes in miRNAs levels in the cells of the eye tissues. This may be a clue for future indications of biomarkers based on circulating miRNAs. Retinal pigment epithelium is the pigmented cell layer between the neural retina and the choroid, and it is required for proper vision [64]. Many researchers claim that modification of this layer is involved in the pathophysiology of DR. Shao et al. indicated that miR-451a acts as a regulator of retinal pigment epithelium (RPE) function. It might play a pivotal role in the regulation of proliferation and migration in RPE cells [65]. Another study proves that miR-142-5p levels were downregulated in high-glucose-treated human retinal endothelial cells. As a direct target of this miRNA, insulin-like growth factor 1 (*IGF1*) was identified. The authors of this article claim that miR-142-5p participates in the progression of DR [66].

#### 2.1.2. Diabetic Nephropathy (DNP)

Late complications of T2DM can also be observed in the kidneys. DNP is characterized by albuminuria and chronic loss of renal function as a result of microvascular failure due to prolonged hypoglycemia [67]. The leading cause of kidney failure is diabetic kidney disease. It is considered as a major kidney-related complication of both types of diabetes mellitus (T1DM and T2DM) and as leading causes of end-stage renal disease (ESRD) [68]. Reports state that miRNAs play a role in the regulation of processes related to kidney failures, such as fibrosis, podocyte apoptosis, mesangial cells proliferation, extracellular matrix accumulation, inflammation, and oxidative damage [69]. Microalbuminuria may be an indicator of early DNP, and macroalbuminuria is related to the progression of this disease [70]. It is crucial to avoid the serious consequences of this disease. MiRNAs seems to be a good prognostic tool for the detection of diabetic kidney failure [71]. One of the most remarkable studies indicated that the level of miR-29a in patient urine can be a predictive biomarker for DNP. The level of this miRNA was significantly upregulated in patients with albuminuria compared to normoalbuminuric individuals [72]. Levels of other studied miRNAs (miR-29b and miR-29c) did not show a significant difference between tested groups. Subsequent studies showing the potential of miRNA as biomarkers for DNP are summarized in Table 3.

A previous study on miR-192 showed that extracellular vesicles (microvesicles and exosomes) isolated from urine could help with the diagnosis of initial stages of DNP. This study showed that levels of miR-192 from urinary extracellular vesicles in T2DM patients with different degrees of albuminuria are increased in the microalbuminuric group compared with the normoalbuminuric subjects and decreased in the macroalbuminuric group. Calculations of the AUC for miR-192 showed a high diagnostic value (AUC = 0.802) in discriminating the normoalbuminuric from the microalbuminuric group [73]. A larger study including 591 patients showed that serum levels of miR-192 in patients of the large albuminuria group were lower (the lowest) than in the microalbuminuria and control groups. It was also lower in the microalbuminuria group than in the healthy control group. This study showed that lower levels of miR-192 were connected with the decrease in the urine albumin ratio and nephritic fibrosis in DNP patients [74]. In a study with over 200 patients with DNP, the authors found that increased miR-196a in urine is associated with progression of renal abnormalities and might be a good prognostic biomarker for renal fibrosis in the patients with DNP [75]. Circulating miR-192 and also miR-377 from blood can serve as potential biomarkers for early detection of DNP. MiR-377 showed increased expression and miR-192 decreased expression in T2DM patients with and without DNP compared with healthy controls. Furthermore, miR-377 and miR-192 were directly associated with albuminuria and showed the ability to distinguish patients with microalbuminuria/macroalbuminuria from those without this state. In discriminating the normoalbuminuric group from the microalbuminuric/macroalbuminuric groups, the AUC was 0.71 for miR-377 and 0.70 for miR-192. Due to the relatively small number of subjects (*n* = 85), further studies taking into account the usefulness of these miRNAs as potential biomarkers for DNP detection are recommended [76]. Kim et al. identified a unique profile of circulating exosomal miRNAs, which may serve as an indicator of DNP in patients with DM [77]. RNA sequencing showed upregulation of serum exosomal miR-1246, miR-642a-3p, let-7c-5p, miR-1255b-5p, let-7i-3p, miR-5010-5p, and miR-150-3p in a group of diabetic patients compared to healthy controls. MiR-4449 was overexpressed in patients with DNP in comparison to a group of patients with DM without this pathological condition. These miRNAs are promising biomarkers for the diagnosis of DNP, and further exploration is needed. The study includes 180 patients with T2DM (90 with ESRD and 90 euglycemic diabetes individuals) and showed that changes in expression of miR-499a are possibly involved in ESRD pathogenesis. The level of miR-499a was decreased in patients with ESRD compared to diabetic individuals without this condition. This difference did not show statistical significance but showed significant correlation with serum MALAT1 (metastasis-associated lung adenocarcinoma transcript 1) and with clinical findings [78]. MALAT1 is a lncRNA (long noncoding RNA) that is able to regulate genes involved in the cycle of cells and is associated with many types of cancers and with microvascular complications of diabetes in mice. Levels of serum MALAT1 were significantly higher in diabetic patients with ESRD than those in the control group. These findings might highlight the importance of further studies on miR-499a. Due to the bioinformatic examination, we are able to observe the relationships between miRNAs and target genes. Assmann et al. in their systematic review and bioinformatic analysis showed six dysregulated miRNAs (miR-21-5p, miR-29a-3p, miR-126- 43 3p, miR-192-5p, miR-214-3p, and miR-342-3p) in DNP individuals in comparison to controls. This study showed that these miRNAs with their targeted genes are involved in processes such as apoptosis, fibrosis, and extracellular matrix accumulation, which are important in pathways of known relevance for DNP pathogenesis [79]. The possibility of linking the miRNA–gene signaling pathways and the identified regulatory network of key miRNA–genes has indicated the crucial role of miR-29 and miR-200 in DNP. Such findings may provide a new idea for further studies focusing on these miRNAs as potential biomarkers [80]. An interesting meta-analysis of profiling studies on humans and animal models of diabetic nephropathy identified three upregulated miRNAs (miR-21-5p, miR-146a-5p, and miR-10a-5p) and two with lower expression (miR-25-3p and miR-26a-5p) compared to controls [81]. These miRNAs required further attention in future studies.

#### 2.1.3. Diabetic Neuropathy (DN)

The cause of DN is a high concentration of glucose in the blood, which results in the formation of glycation end-products that cause changes in the nerves (such as demyelination) or the nerve fibers themselves. It is associated with sensory neuronal damages and neuropathic pain. It can increase the risk of infections, foot ulcers, and nontraumatic amputation. Two main types of neuropathy are distinguished: that involving the peripheral sensorimotor and that involving the autonomic nervous system [82,83,84]. Many studies report that miRNAs are extremely important in the nervous system as a regulator of gene expression. MiRNAs have long been known to play an essential role in processes such as neurogenesis, neuron survival, dendritic outgrowth, and spine formation [85]. Changes in miRNA levels might be relevant to disorders such as DN [86]. In comparison with other microvascular complications, there are not many studies in the literature considering miRNAs as a potential biomarker for DN. Most of the research is carried out on animal models. The topic of miRNA in DN undoubtedly requires further investigation based on the material from diabetic patients suffering from this condition.

It has been observed that diabetes in rats is responsible for reduced expression of miR-146a in static nerves. The changes in its expression are related to the inflammatory responses that intensify nerve damage [87]. In another study, miR-146a showed downregulation in sciatic nerve tissue in mice. Furthermore, the authors claimed that treatment with miR-146a mimics elevated miR-146a levels in plasma and reduces the neuropathy [88]. An interesting study on mice models showed that miR-190a-5p is involved in DN through targeting *SLC17A6* (solute carrier family 17 member 6) [89]. In this study, the authors observed decreasing level of miR-190a-5p and at the same time increased expression of *SLC17A6* in the spinal tissue of mice with neuropathy compared to controls. *SLC17A6* plays a key role in synaptic transmission in the nervous system. Such findings can provide a new perspective for the treatment of this disease. Further research on circulating miR-190a-5p is needed to determine the possibility of this biomarker for DN in humans. Zhang et al. suggest that miR-25 might be a good diagnostic and therapeutic target for DN. Their study gave pieces of evidence for reduced miR-25 expression on sciatic nerves in diabetic mice [90].

The abovementioned miR-146a was also tested in human samples. Research based on sequencing claims that polymorphisms in miR-146a and miR-128a gene are associated with DN [91]. In white blood cell fraction, expression of miR-21, miR-146a, and miR-155 is altered in patients with peripheral neuropathies of different origins [92]. Ciccacci et al. in their study showed that miR-499a polymorphism might be associated with DN tendency [93]. Polymorphism studies indicated the crucial role of these miRNAs in the development of DN.

MiR-199a-3p is considered a pro-angiogenetic factor [94]. Li et al. indicated elevated expression of plasma miR-199a-3p in patients with T2DM and elevated expression of this miRNA from lower limb skin tissues in a group of patients with DN compared to a control group. Furthermore, the research shows that overexpression of this miRNA inhibits the expression of strepin E2 (extracellular serine protease inhibitor E2). It was observed that miR-199a-3p is associated with the progression of DN by lowering the level of strepin E2. The authors also suggest that regarding miR-199a-3p, further research should focus on the potential role of this miRNA as a biomarker for the detection of DN, but also as a new therapeutic target for the treatment of this chronic complication [95]. Another study indicated increased miR-199a-3p levels in the group of patients observed in ectosomes of T2DM patients compared to healthy controls [96]. Another interesting study used weight correlation network analysis and identified genes and signaling pathways related to DN. For miR-377, miR-216a, and miR-217 associated with T2DM, targeted genes were predicted, which should be considered as putative biomarkers for progression of the disease [97].

Poorly controlled diabetes, prolonged hyperglycemia, and damaged nerves can lead to diabetic foot and diabetic foot ulcers [45]. Healing of the foot wound is important in the treatment of foot ulcers and the prevention of lower limb amputation. Researchers have stated that endothelial progenitor cells from bone marrow have a crucial role in angiogenesis and functions of vascular endothelial cells. Reduced levels of these cells in foot lesions are observed [98]. Gao et al. proved that miR-155 is overexpressed in endothelial progenitor cells (EPCs) from patients with diabetic foot ulcers. Moreover, the expression of this miRNA was also elevated in high glucose-induced EPCs from healthy people. MiR-155 takes part in the regulation of crucial signaling pathways connected with angiogenesis, proliferation, and wound healing [99]. Other remarkable findings pointed out a crucial role of miR-203. Liu et al. proved that this miRNA in the human skin tissue sample can serve as a new biomarker for early diagnosis for the severity of diabetic foot ulcers. As miR-203 can play a pivotal role in diabetic wound healing, further research should be carried out taking into account this miRNA for easier detection of DN [100].

Undoubtedly, there is a lack of studies that examine miRNAs in biofluids associated with DN in humans. There is a need for further investigation to emphasize specific circulating miRNAs as potential biomarkers for DN and therapeutic targets for this pathological condition.

### 2.2. Macrovascular Complications

Atherosclerosis is the main mechanism responsible for the appearance of macrovascular complications. Atherosclerosis is the result of chronic inflammation and damage to the arterial walls. This disease leads to a narrowing lumen of arteries in the whole body. The cause of the narrowing is atherosclerotic plaque, mainly made of deposits of calcium and fatty lipids, which grows from the artery wall, causing a decline in blood flow and resulting in hypoxia of the organs [101,102]. Diabetes accelerates all atherosclerotic lesions by intensifying inflammatory processes, as a result of which the plaque increases. The plaque gradually narrows the diameter of the coronary vessels. These pathological changes in diabetic patients might result in coronary artery disease (CAD), myocardial infarction (MI), peripheral artery disease (PAD), and cerebrovascular disease [42,102].

#### 2.2.1. Coronary Artery Disease (CAD) and Myocardial Infarction (MI)

It is considered that cardiovascular disease and especially CAD are the leading reasons for morbidity and mortality in DM patients [11,12]. CAD typically occurs when a coronary artery develops atherosclerosis [103]. A different degree of artery obstruction is the main cause of imbalance between blood supply and oxygen demand in myocardial cells, which is a characteristic feature of this disease. This condition is also related to reduced availability of nutrients and insufficient removal of metabolic products [104]. It often leads to heart muscle damage and MI [105]. In most cases, MI is caused by an acute clot closing the lumen of the coronary artery supplying blood to the heart. MI can cause the cardiac remodeling and the development of chronic heart failure and is considered as the leading cause of death [106].

It is well understood that miRNAs have a huge impact on cardiovascular biology. Differences in miRNAs expression have been described in many cardiac cases, including CAD and MI [107,108]. MiRNA also seems to be usable in predicting cardiological complications of diabetes. A recent, well-designed study examined the miRNA profiles in diabetic patients suffering from heart disease and in DM patients free from complications. Preferably, such results should be compared to miRNA levels in healthy people, but also in people with heart disease without T2DM.

Kumar et al. identified two plasma miRNAs that can have a pivotal role in the early prediction of CAD. The authors claimed that that miR-133b and miR-21 have different expression in patients with CAD in comparison to healthy controls. MiR-133b showed underexpression and miR-21 overexpression in the studied groups [109]. This work claimed that the group of T2DM patients with CAD did not show any significant differences in the expression of the studied miRNAs. Luo et al. first suggested that plasma miR-30c might be a potential novel biomarker for diagnosis, treatment, and prognosis of CAD in T2DM patients. Their study showed that the levels of circulating miR-30c were remarkably lower in groups of patients with T2DM and T2DM with CAD in comparison with CAD patients and the control group. Diabetic patients with CAD showed the lowest level of miR-30c. Patients from the CAD T2DM group showed a significant negative correlation between circulating miR-30c levels and the degree of coronary lesion severity (*r* = −0.7817, *p* < 0.0001). The authors also postulated that miR-30c takes part in the regulation of plasminogen activator inhibitor 1–vitronetin interactions. The ROC analysis showed high values of the AUC for miR-30c as a diagnostic biomarker for CAD, T2DM, and T2DM, with CAD showing AUC values close to 0.9, which can distinguish these groups from healthy controls. However, the ROC analysis showed that miR-30c can differentiate CAD T2DM patients from diabetic patients without complications only with an AUC of 0.685 [110]. Seleem et al. indicated that serum miR-342 and miR-450 might be important indicators of CAD in T2DM patients [111]. It was found that miR-342 was significantly overexpressed in T2DM, CAD, and T2DM with CAD groups when compared with the control group. In the T2DM with CAD group, the expression was higher than in the CAD group. MiR-450 showed significantly lower expression in the studied group compared to controls. In the group of T2DM with CAD, the expression was significantly lower than in the CAD group. Additionally, according to chi-squared analysis of the T2DM group and the T2DM with CAD groups, these miRNAs demonstrated potential as bioindicators for CAD as a complication of T2DM. MiR-342 reached an AUC of 0.781, whereas miR-450 had an AUC of 0.824. A new, interesting study displayed that serum miR-1 and miR-21 have a crucial role in the diagnosis of CAD. Serum miR-1 levels were significantly lower in patients with T2DM when compared to the healthy control group. Moreover, the authors found out that the expression was the lowest in the group of T2DM patients with heart failure. In contrast, serum miR-21 levels were higher in T2DM patients compared to healthy individuals. The expression was highest in the group of diabetic patients with heart failure. ROC analysis showed that miR-21 might be a novel biomarker in the prediction of heart failure in diabetes patients [112]. These two miRNAs were previously described as related to heart hypertrophy in hypertensive patients. This proves that expression profiles in patients with hypertension differ to those in patients in healthy control groups [113]. Plasma miR-126 and miR-210 expressions differ in patients with diabetes and CAD to those of healthy controls. MiR-210 showed reduced levels in diabetes but also in diabetes with CAD. In contrast, levels of miR-126 were higher in T2DM patients and increased further in those with diabetes with CAD. Both miRNAs presented high diagnostic values in distinguishing T2DM patients with and without CAD from healthy controls. These findings suggest that these miRNAs might serve as potential markers for CAD as a complication of T2DM [114]. It is worth considering that miR-126 was also tested for different diabetes complications (DR and DNP) mentioned in this review. To determine the exact specificity of this miRNA for diabetes complications, further studies need to be conducted on a larger group of subjects. Interestingly, miR-126 and miR-26a from circulating microparticles are considered as associated with risk of CAD. Levels of these miRNAs were significantly lower in T2DM patients than in healthy controls. Decreased expression levels of miR-26a and miR-126 are related to the coexistence of the CAD [115]. Diabetic cardiomyopathy is diagnosed when ventricular myocardial dysfunction develops in patients with diabetes, even if CAD or hypertension has not developed. It is primarily caused by metabolic disorders in diabetes due to insulin deficiency. Accumulation of lipids in the myocardium is considered a hallmark of diabetic cardiomyopathy. Another study suggests that serum miR-1 and miR-133a might be potential biomarkers for early diagnosis of diabetic cardiomyopathy [116]. A good direction for future research might be considering miR-483-3p as a potential prognostic biomarker for cardiovascular diseases in T2DM patients. This miRNA is a crucial regulator of endothelial integrity. Furthermore, the authors claimed that miR-483-3p might be a potential therapeutic target [117].

There are not many studies demonstrating differences in miRNA profiles in human suffering from diabetes and macrovascular complications and those free from complications. However, many works describe the important role of miRNA in the development of diseases such as CAD, MI, and hypertension without a T2DM group. MiRNA such as miR-21, miR-155, miR-126, miR-146a/b, miR-143/145, miR-223, and miR-221 are most frequently described in hypertension and atherosclerotic [118]. The presented miRNAs were also indicated as significant in patients with diabetes. Research showed that miR-223 is associated with the severity of CAD. The AUC for this miRNA reached 0.933, which indicates a promising ability to differentiate CAD cases from healthy controls. Expression of this miRNA was higher in the group of CAD individuals than in the control group. The level of miR-223 increased with increasing severity of the disease. This shows evidence that this miRNA could be a good potential biomarker in the assessment of CAD. However, the authors did not separately analyze the group of patients with diabetes [119]. Interestingly, it has been proven that miR-223 is upregulated in the blood of patients with T2DM [120].

Wang et al. in a meta-analysis study showed that the level of serum miR-133a-3p was upregulated in samples of patients with CAD. Additionally, this study suggested that miR-122-5p might be a valuable biomarker for this disease [121]. This study confirmed the earlier report about the importance of this miRNA in the pathology of the cardiovascular system [122]. MiR-122-5p was also previously described to have a crucial role in atherosclerosis and CAD [123,124]. These reports suggest that miR-122 and miR-133a should be considered as potential biomarkers for cardiovascular events and should be also tested for diabetes complications. Serum exosomal miR-1915-3p, miR-4507, and miR-3656 might be novel diagnostic tools for early-stage acute myocardial infarction [125]. The expression of these miRNAs was significantly lower than in the stable CAD sample exosomes. ROC analysis indicated that miR-1915-3p and miR-3656 had the highest diagnostic value (AUC > 0.77), which suggests that this miRNA can be a predictive tool for acute MI. Expression of miR-21, miR-155, and miR-221 in peripheral blood mononuclear cells (PBMCs) was significantly different among patients with CAD and controls. MiR-21 showed overexpression, whereas miR-221 and miR-155 were downregulated in the studied group [126]. It is worth noting that these miRNAs have a crucial role in the pathogenesis of diabetes and were also indicated as potential biomarkers in microvascular complications as well as in T2DM patients with CAD [55,92,127,128]. Changes in these miRNAs seem to be typical for vascular inflammation, and to use them as potential biomarkers, more studies with larger cohorts are necessary. Serum miR-584-5p was recently considered as a potential biomarker for CAD. It is observed that in patients with CAD, expression of this miRNA is lower than in patients without this disease. However, the groups were relatively small, and the authors of the study did not identify a group with T2DM [129]. A recent study from 2020 reports that the combination of four plasma miRNAs (let-7i-5p, miR-32–3p, miR-3149, and miR-26a-5p) has good diagnostic value (AUC = 0.837) for distinguishing patients with severe CAD from controls [130]. While the mentioned studies did not include patients suffering from diabetes, further research should be directed to these miRNAs as potential biomarkers, indicating a group of diabetics with a predisposition to developing specific macrovascular complications.

#### 2.2.2. Peripheral Artery Disease (PAD)

Peripheral artery disease is considered a group of diseases affecting the peripheral arteries that lead to narrowing or blockage of the large arteries, except for coronary arteries, aortic arch, and brain arteries [131,132]. PAD plays a role as a predictor of MI, stroke, and death due to vascular abnormalities [133]. A big challenge for the diagnosis of this condition is the fact that a lot of patients are living without symptoms, causing slow progression of the disease [134]. Diabetic patients may remain asymptomatic due to the often associated neuropathies [131]. The circulating miRNA has been connected with PAD in a number of studies [135,136,137]. Numerous pieces of evidence shows that miRNAs are involved in the regulation of many key processes connected with the pathogenesis of PAD, such as angiogenesis, inflammation, and endothelial function [138]. Signorelli at al. in their pilot study indicated serum miR-130a, miR-27b, and miR-210 as potential good biomarkers for PAD. The expression of this miRNA was significantly increased in PAD patients versus healthy controls. In this case, about 37% of PAD group had diabetes [139]. There is also research focused on miRNA as a potential marker for the advanced stage of PAD, which is critical limb ischemia. Plasma miR-4739 levels are significantly elevated in the group of T2DM patients with critical limb ischemia in comparison to T2DM individuals without this complication [140]. Similarly, serum miR-323b-5p seems to have a great diagnostic value in distinguishing patients with critical limb ischemia in T2DM groups [141]. Both miR-4739 and miR-323b-5p have not been previously studied in the context of PAD. Studies are showing an enormous role of miRNA in the pathogenesis of PAD. Most of them are based on animal (miR-93, miR-92a, miR-503, and miR-100), cell cultures (miR-221, miR-126, and miR-1), or human tissue studies (miR-15a, miR-126, miR-223, miR-28-3p, miR-21, and miR-503) [142]. There is a need to select fluid biomarkers for humans. More research is required to find the right, reliable biomarker for the early diagnosis of PAD in people with diabetes. The current state of knowledge allows scientists to focus their efforts on intensified research on larger groups of patients.

#### 2.2.3. Cerebral Vascular Disease and Stroke

Particular attention should be paid to the search for markers in body fluids that can help indicate predisposition to developing cerebral complications among T2DM patients. Cerebral vascular disease including stroke is categorized as a macrovascular complication of diabetes. Unfortunately, such complications are more severe and require more attention during the treatment of patients with diabetes in comparison to patients without it. Individuals with DM are more likely to develop cerebrovascular disease and strokes [143]. The main reason for these complications is atherosclerosis [143,144]. Cerebrovascular disease refers to medical conditions in which blood flow to specific parts of the brain is impaired. Vascular diseases often lead to a stroke. These changes are not, unlike microangiopathy, due to a disease associated with diabetes only and might also occur without any relation to diabetes. However, the coexistence of diabetes significantly increases the course of the atherogenic process and worsens the prognosis. [145]. Stroke was determined to be the second leading cause of death after CAD in 2015 [146]. There are two main types of stroke: ischemic and hemorrhagic. An ischemic stroke occurs when an artery that supplies the brain with blood is blocked. The most common cause is the enlargement of atherosclerotic plaque, which leads to obstructed blood flow to the brain. Hemorrhagic stroke is a consequence of the rupture of the cerebral artery wall and bleeding outside the vessel [147,148]. Diabetes is a risk factor for both types of strokes, and patients with diabetes are at a much higher risk of stroke than those without it [149,150,151,152].

Comparatively, like in other diabetes complications, miRNA plays a crucial role in risk factors of the cerebral dysfunctions. As of 2009, circulating miRNAs began to be viewed as potential stroke biomarkers [153]. In a recent meta-analysis conducted in 2020, the authors pointed out the crucial role of miR-320b and miR-320d in the pathogenesis of stroke [154]. Interestingly, these two miRNAs were previously described as biomarkers related to diabetes [155,156]. MiR-320b was also described as a potential indicator of carotid atherosclerosis [157]. Many studies present miR-21 as a biomarker in different types of strokes. However, this particle seems to be typical not only of strokes, but also of many diseases, including DM and its other complications [54,158].

Plasma and platelet miRNAs also play a crucial role in the development of complications related to diabetes. In their work from 2014, the authors of [159] indicated significant downregulation of platelet and plasma miR-223 and miR-146a in patients with T2DM and ischemic stroke or only with T2DM compared to HC. This expression was not downregulated in patients with ischemic stroke only. This study suggests that platelet and plasma miR-233 and miR-146a could be specific markers for T2DM with or without ischemic stroke. However, this study should be repeated on a larger number of patients. MiR-223 from blood was previously described as a marker for acute ischemic stroke. In a study by Wang et al., the authors compared patients with ischemic stroke with healthy controls, where 39.2% of patients had diabetes. The level of this miRNA was increased in the group with ischemic stroke compared to controls. The authors of [160] also suggest that IGF-1 might be a new target for miR-223. Long et al. investigated the expression of miR-223 from PBMCs in patients with cerebral infarction. This study also had a group of patients with T2DM and stroke. Expression of this miRNA was lower in patients with T2DM than in HC. There was no significant difference between the expression of this miRNA when comparing patients with cerebral infarction or cerebral infarction with T2DM to healthy controls [161]. One of the better-designed studies shows that plasma and platelet levels of miR-223 and miR-144 may serve as biomarkers associated with ischemic stroke in T2DM patients. The miR-144 levels were significantly higher in T2DM patients with ischemic stroke than in healthy controls and the T2DM group. In contrast, levels of miR-223 were lower in the group of patients with T2DM and ischemic stroke. It is suggested that these altered expressions of this miRNA increased susceptibility to ischemic stroke in T2DM [162]. Serum miR-503 could be a good biomarker for ischemic stroke in diabetic patients. In recent research, the authors of [163] observed overexpression of this miRNA in a group of DM patients with ischemic stroke when compared to nondiabetic patients with stroke, DM patients, and healthy controls.

## 3. Conclusions

To avoid long-term vascular complications associated with T2DM, scientists must pay attention to early diagnosis. MiRNAs play an extremely important role in many processes leading to the development of T2DM and related complications. These molecules have substantial potential as biomarkers for vascular complication of T2DM, as evident in the growing body of research data, mainly due to their high specificity and sensitivity. Recent studies show their identification mostly in serum; however, in further studies, it is worth noting the examination of miRNA signatures in other body fluids, such as saliva or urine. Almost all of the work mentioned above requires continuation and further research on a much larger group of people, as well as validation of the results by other, standardized methods. However, it is clear that, despite some limitations, the identification of new miRNAs offers a promising perspective for future functional research related to the development of complications in T2DM patients.

## Figures and Tables

**Table 1:** Main complication of diabetes mellitus.

Complication of Diabetes			
Acute	Chronic		
diabetic ketoacidosis	**Vascular**		**Nonvascular**
nonketotic hyperosmolar coma	**Microvascular**	**Macrovascular**	sexual dysfunctions
hypoglycemia	retinopathy	coronary artery disease	skin complications
diabetic coma	nephropathy	peripheral artery disease	
	neuropathy	cerebrovascular disease including ischemic stroke	

**Table 2:** Latest reports concerning miRNAs in biofluids of human patients with DR.

miRNA	Sample Type	Expression in Research Group vs. Controls	Number of Cases	Method	Significant Findings	Year of Publication	References
miR-126	serum	down in T2DM patients compared with control group	*n* = 186; 100 T2DM (14 without complications, 26 with macrovascular complications, 17 DN, 24 DNP, 19 DR), 86 IGT	qPCR	serum miR-126 expression might serve as a potential biomarker for DR	2016	[50]
	serum	down	*n* = 184; 125 DM (44 NDR, 42 NPDR, 39 PDR) and 59 HC	qPCR	high values of AUC in ROC analysis determine miR-126 as a good diagnostic biomarker that differentiates PDR patients from HC	2017	[51]
miR-200b	serum	down	*n* = 508; 255 DR, 253 HC	qPCR	miR-200b targets *VEGFA* gene	2017	[52]
miR-93	plasma	up	*n* = 267; 140 T2DM (75 DR, 65 NDR) 127 HC	qPCR	levels of plasma miR-122 might serve as DR biomarkers	2017	[53]
miR-21	plasma	up	*n* = 304; 65 NDR, 73 NPDR, 51 PDR, 115 HC	qPCR	elevated miR-21 expression can be used to identify occurrence and stage of DR	2017	[54]
miR-221	serum	up (progressively upregulated in NDR, NPDR, and PDR)	*n* = 134; (33 HC, 37 NDR, 34 NPDR, 30 PDR)	qPCR	miR-221 might serve as a biomarker for progression and occurrence of DR	2018	[55]
let-7a- 5p miR-28-3p miR-novel-chr5_15976	serum	up	screening phase: 9 (3 T2DM NDR, 3 T2DM DR, 3 HC); training phase: 20 (10 T2DM NDR, 10 T2DM DR); validation phase: 79 (29 T2DM-DR, 50 T2DM NDR)	RNASeq, qPCR	this miRNA signature may serve as a biomarker for DR; better than single miRNA	2018	[56]
miR-423	serum	down in PDR	*n* = 69; (22 HC, 10 T2DM NDR, 22 NPDR, 15PDR)	qPCR	miR-423 may serve as a biomarker for DR; is correlated with *VEGF*, *NO,* and *eNOS* expression	2019	[57]
miR-122	serum	up in T2DM NDR and T2DM with NPDRdown in T2DM PDR	*n* = 40; (10 of HC, 10 of T2DM NDR, 10 of T2DM with NPDR, 10 of T2DM with PDR	qPCR	levels of miR-122 in serum of T2DM patients might determine occurrence and progression of DR	2019	[58]
miR-29b, miR-200b	plasma	down	*n* = 206; 186 T2DM (91 NDR, 46 NPDR, 49PDR), 20 HC	qPCR	downregulation of miR-29b is associated with progression of DR	2019	[59]
miR-4448, miR-338-3p, miR-190a-5p, miR-485-5p, miR-9-5p	serum	down: miR-4448, miR-338-3p, miR-485-5p, and miR-9-5pup: miR-190a-5p	*n* = 21; 10 NPDR, 11 NDR	RNASeq	these miRNAs might serve as good potential biomarkers for DR with high AUC value (0.909)	2019	[60]
miR-3197, miR-2116-5p	serum	up	*n* = 90; 42 NPDR, 3 PDR, 45 NDR	microarray, qPCR	high diagnostic value of these 2 miRNAs can indicate patients with DR; *NOTCH2* as a possible target gene of miR-2116-5p	2020	[61]
miR-320a	plasma	down	*n* = 170; 60 HC, 48 DM without DR, 62 DR	qPCR	DR can be identified by plasma miR-320a measurement; *TSC1* and *CDK6* are possible target genes for this miRNA	2020	[62]
let-7b, miR320b, miR-762, miR-4488	aqueous humor, plasma, vitreous	miRNA let-7b—up in aqueous and vitreous, down—plasma miR-320b—up in aqueous, vitreous, and plasma miR-762 and miR-4488—up in vitreous; up in PDR, down in NPDR in aqueous; down in PDR and up in NPDR in plasma	*n* = 27; 11 HC, 16 DM: 5 T1DM PDR, 7 T2DM PDR and 4T2DM NPDR	microarray, qPCR	this miRNA signature may contribute to the diagnostic tests or therapeutic approaches for the DR	2020	[63]

AUC: area under curve; DR: diabetic retinopathy; DN: diabetic neuropathy; DNP: diabetic nephropathy; eNOS: endothelial nitric oxide synthase; HC: healthy controls; IGT: impaired glucose tolerance; NDR: no diabetic retinopathy; NO: nitric oxide; NPDR: non-proliferative diabetic retinopathy; PDR: proliferative diabetic retinopathy; qPCR: quantitative polymerase chain reaction; RNASeq: RNA sequencing; ROC: receiver operating characteristic curve; T1DM: type 1 diabetes mellitus; T2DM: type 2 diabetes mellitus; VEGF: vascular endothelial growth factor.

**Table 3:** Latest reports concerning miRNAs in biofluids of human patients with DNP.

miRNA	Sample Type	Expression in Research Group vs. Controls	Number of Cases	Method	Significant Findings	Year of Publication	References
miR-192, miR-194, miR-215	urinary EVs	up in microalbuminuria patients	*n* = 90; 80 T2DM (30 normoalbuminuric, 30 microalbuminuric, 20 macroalbuminuric) 10 HC	qPCR	miR-192 has the highest diagnostic value (AUC = 0.802); miR-192 and miR-215 levels are positively correlated with TGF-β1 levels	2016	[73]
miR-192	serum	down	*n* = 591; 464 T2DM (157 normal albuminuria, 159 microalbuminuria, 148 large albuminuria), 127 HC	qPCR	lower level of miR-192 is connected with the decrease in urine albumin ratio; miR-192 has potential for DNP diagnosis	2016	[74]
miR-196a	urine	up	*n* = 209 T2DM DNP	qPCR	miR-196a is a good candidate for a noninvasive marker for the progression of renal fibrosis in DN patients	2018	[75]
miR-192, miR-377	whole blood	miR-377—up, miR-192—down	*n* = 85; 55 T2DM (30 without DN, 15 microalbuminuric, 10 macroalbuminuric), 30 HC	qPCR	both miRNAs can serve as a potential biomarker for DNP and are correlated with DNP risk factors	2018	[76]
miR-1246, miR-642a-3p, let-7c-5p, miR-1255b-5p, let-7i-3p, miR-5010-5p, miR-150-3p, miR-4449	serum exosomes	up	*n* = 74; 18 HC, 33 DM without DNP, 23 DNP	RNASeq	presented miRNAs are correlated with the albuminuria degree and might be helpful for diagnosis of DNP	2019	[77]
miR-499a	serum	down	*n* = 180; 90 T2DM with ESRD; 90 T2DM without ESRD	qPCR	altered expressions of miR-499a are possibly involved in DNP, and its level is correlated with serum MALAT1	2018	[78]

AUC: area under curve; DNP: diabetic nephropathy; EVs: extracellular vesicles; ESRD: end-stage renal disease; HC: healthy controls; qPCR: quantitative polymerase chain reaction; RNASeq: RNA sequencing; T2DM: type 2 diabetes mellitus.
